# Continuous Updating via Self-Motion Compensates for Weak Allocentric Spatial Memory in Aging

**DOI:** 10.1037/pag0000926

**Published:** 2025-08-11

**Authors:** Andrea Castegnaro, Alexander Dior, Neil Burgess, John King

**Affiliations:** 1 Institute of Cognitive Neuroscience, University College London; 2 Queen Square Institute of Neurology, University College London; 3 Department of Clinical, Educational and Health Psychology, University College London

**Keywords:** spatial memory, self-motion, spatial updating, virtual reality, healthy aging

## Abstract

Navigational skills are essential for interacting with our environment, supported by multiple types of spatial representations. We investigated age-related differences in spatial memory using a virtual reality task that manipulated viewpoints between the encoding and retrieval of one or four-object locations. The task investigates compensatory mechanisms in aging, specifically how spatial updating via self-motion affects spatial memory. We tested 21 young adults (ages 19–36) and 23 older adults (ages 63–80). The task involved three movement conditions: same-viewpoint condition, where participants walked away and returned to the same viewpoint; shifted-viewpoint (walking) condition where participants walked to a different viewpoint, enabling continuous updates of their egocentric representations through self-motion; and shifted-viewpoint (teleport) condition where participants teleported to the other viewpoint, involving both a virtual translation and rotation of the participant’s view. Retrieval was tested by asking participants to place each object at its previously seen location. Average displacement error was affected by age group, object configuration, and movement condition, with an interaction between age and movement condition. Differences in movement conditions were primarily driven by older participants, who were most accurate from the same viewpoint. In shifted-viewpoint conditions, teleportation—where self-motion cues were absent—led to significantly greater errors than walking in the older group. Our results highlight the role of spatial updating in supporting spatial memory and suggest that age-related decline in allocentric representations can be mitigated by continuous updating of egocentric representations by self-motion. We speculate that the use of spatial updating might be impaired early in the progression to Alzheimer’s dementia due to entorhinal cortical pathology.

Navigational skills are essential for interacting with our environment, supported by the ability to form spatial representations. Humans and other mammals utilize two types of spatial representations: egocentric, which locates objects in a framework based on the observer’s location, and allocentric which involves interrelationship among environmental elements independent of the observer’s location (Banta Lavenex et al., 2011; Burgess, 2006; for a review see Colombo et al., 2017). These representations are largely relying on different neural substrates, with egocentric strategies mainly associated with the parietal lobe and subcortical structures such as the striatum (Whitlock et al., 2008), while allocentric strategies are primarily associated with the medial temporal lobe, including the hippocampus (Burgess et al., 2002; Ekstrom et al., 2014), entorhinal cortex (Alexander et al., 2023; McNaughton et al., 2006), and retrosplenial cortex, which plays a key role in transforming allocentric spatial information into egocentric representations (van Wijngaarden et al., 2020).

Egocentric representation is continuously refined by spatial updating—the online integration of self-motion signals from vestibular, proprioceptive, motor-efference copy, and optic flow—to keep track of how the positions of environmental constituents such as objects and landmarks change as we move (Klatzky et al., 1998; R. F. Wang, 2016). Allocentric representation is preserved by anchoring relational codes to stable environmental landmarks and boundary geometry, thereby maintaining the metric distances and orientations among cues over time that are independent from the observer’s location (Byrne & Crawford, 2010). Spatial updating can operate in both egocentric and allocentric representations. In egocentric representations, each new displacement is simply subtracted from the current egocentric vectors. In an allocentric representation, however, the same self-motion vector must first be converted into world-centered coordinates before it can be used to update the observers’ position within a stable external map, making the process inherently more complex than in the egocentric case (Frances Wang, 2017; Rolls, 2020). The self-motion signals that maintain egocentric accuracy therefore also contribute to the incremental construction and recalibration of allocentric maps, supported by the hippocampal–entorhinal neural substrate (McNaughton et al., 2006). Conversely, allocentric configurations can also be derived in the absence of physical movement when rich visual or contextual information specifies the spatial relations among environmental constituents (Ekstrom et al., 2014). In this framework spatial updating and allocentric processing are complementary: updating keeps egocentric codes synchronized with the external world in real time, while established allocentric maps provide a stable scaffold that can reset the updating process after disorientation (Burgess, 2006; Frances Wang, 2017).

Age-related deficits in spatial processing typically manifest first in allocentric strategies, usually tested by encoding constituents of an environment from one viewpoint and retrieving them from another (Bohbot et al., 2012; Lithfous et al., 2014), with egocentric deficits appearing later (Gazova et al., 2013; Lopez et al., 2019). These deficits have been consistently associated with hippocampal atrophy (Driscoll et al., 2003; Raz et al., 2005) and hippocampal reduced activation (Moffat et al., 2006) occurring with natural aging. Lesion studies further support the role of the hippocampus in viewpoint-shifted spatial memory, showing severe impairments in spatial recall from novel viewpoints in patients with hippocampal damage (Hartley et al., 2007; King et al., 2002). However, recent evidence indicates that spatial memory impairments associated with hippocampal damage and aging may reflect an overall reduced precision in spatial memory rather than an absolute loss of allocentric representations. Patients with hippocampal lesions retain partial allocentric memory abilities in tasks analogous to the Morris water maze but exhibit less precise and more variable spatial responses, characterized by longer and more circuitous paths to the remembered target locations (Kolarik et al., 2018; McAvan et al., 2022). Similarly, older adults demonstrate reduced spatial precision but maintain effective use of allocentric strategies, as indicated by their continued reliance on stable distal landmarks (McAvan et al., 2021). Nevertheless, the field still lacks a clear consensus on what aspects of egocentric and allocentric memory are specifically affected in aging. Possible explanations for these differing findings involve compensatory mechanisms. For instance, proprioceptive and vestibular cues available in immersive virtual reality may help mitigate age-related declines in allocentric memory (Hill et al., 2024). Additionally, older adults may compensate by relying on geometric cues (Bécu et al., 2019; Bian & Andersen, 2013; Rodgers et al., 2012), potentially mediated by the entorhinal cortex’s role in continuously updating spatial representations using self-motion information (Bohbot et al., 2007; Iaria et al., 2009; León et al., 2016). This compensatory capacity suggests a resilience in egocentric processing that may offset allocentric decline.

Given the lack of consensus on age-related differences in spatial representations and the proposed role of compensatory mechanisms, the present study aims to directly investigate these issues. Specifically, we examine how spatial updating via self-motion influences spatial memory performance in younger and older adults. To this end, we developed an immersive virtual reality task, Queen Square Virtual Reality (VR; Figure 1), designed to manipulate participants’ viewpoints between the encoding and retrieval of object locations within an environment (King et al., 2002). A same-viewpoint condition assesses spatial memory after having participants walk away from and return to the original encoding viewpoint, providing a baseline against which the effects of viewpoint shifts can be compared. In a shifted-viewpoint condition, participants walk between viewpoints, enabling them to perform spatial updating continuously through self-motion signals. Finally, we introduced a second shifted-viewpoint condition in which participants actively perform a “teleportation” via a button press, setting both their virtual position and orientation to the new viewpoint, removing self-motion inputs from vestibular, optic flow, and proprioceptive systems that normally drive spatial updating (Etienne & Jeffery, 2004).

In the same-viewpoint condition, we expected minimal age-related performance differences as participants may rely on egocentric representations of object locations such as visual or motoric (e.g., pointing or gaze) directions, which remain relatively intact in normal aging. Conversely, we expected age-related performance differences between the two shifted-viewpoint conditions. Specifically, in the shifted-viewpoint (walking) condition, participants could continuously update egocentric representations using self-motion cues, potentially supporting spatial accuracy despite viewpoint shifts. In contrast, the shifted-viewpoint (teleport) condition removes this continuous spatial updating, compelling participants to rely exclusively on allocentric representations. Thus, we hypothesized greater age-related impairments specifically in the teleport condition compared to the walking condition, reflecting the vulnerability of allocentric spatial representations to aging. Allocentric representations, of how objects are located relative to environmental cues, could support retrieval of object locations in all three conditions. Importantly, allocentric representations are available irrespective of viewpoint shifts or whether self-motion information is present between encoding and retrieval. Consequently, because any performance difference observed between the same-viewpoint and shifted-viewpoint (walking) conditions could be attributed either to impaired spatial updating via self-motion or to weakened allocentric representations, no specific hypothesis was formulated regarding their comparison.

We incorporated two object configurations: one object and four objects. The one-object configuration simplifies the task, allowing us to assess basic comprehension and ability to perform spatial retrieval in various conditions without significant memory load. Conversely, the four-object configuration increases the memory load and, across the three conditions, reveals which types of representation are available to support this increased load. It is also worth noting that shifting viewpoint testing of multiple object locations affords the use of an allocentric representation of the objects compared to mental rotation of a single object around the viewpoint (King et al., 2002). We hypothesized that aging would impact performance in the multi-object shifted-viewpoint conditions, consistent with previous reports of more general age-related declines in working memory capacity (Castillo Escamilla et al., 2023).

## Method

### Participants

A priori power analysis was conducted using G*Power to determine the required sample size for the analysis of variance (ANOVA) mixed design with three within factors and one between factors. Based on prior research, an estimated effect size (Cohen’s *f*) of 0.31 was used (Rodgers et al., 2012). The analysis assumed an α level of .05, power of 0.90, and a correlation of 0.25 among repeated measures. The results indicated a total sample size of 44 participants, with 22 per age group, to detect significant effects. Young participants (*n* = 21; 52% female) between ages 19–36 (*M* = 23.5, *SD* = 4.2) were recruited from the University College London Sona participant pool. Older adult participants (*n* = 23; 52% female) between ages 63–80 (*M* = 72.3, *SD* = 5.20) were recruited through the “Join Dementia Research” online database. Exclusion criteria were the presence of any major medical or psychiatric disorder, elevated anxiety or depression symptoms assessed in a phone interview through the Generalized Anxiety Disorder–2 and Patient Health Questionnaire–2 screening tools (Staples et al., 2019), epilepsy, a history of alcohol excess or any mobility or visual impairment which may compromise performance in immersive virtual reality testing.

Ethical approval for the study titled “Investigating Spatial Memory Functions Using Immersive Virtual Reality (iVR)” was granted by the University College London Research Ethics Committee (ID No. SHaPS-2018-JK-027). Ethics were in line with the regulations outlined in the Declaration of Helsinki.

### Queen Square Virtual Reality Task

The Queen Square VR task was administered using the HTC Vive PRO VR system, equipped with a wireless adapter to avoid cable-related issues, tracking an area of 10.0 × 5.0 m^2^ for free movement. The VR environments were developed in Unity3D (Unity Software, Inc.) and Autodesk Maya (Autodesk Inc.). The entire task took approximately 50 min to complete.

### Training Phase

**Figure 1:**
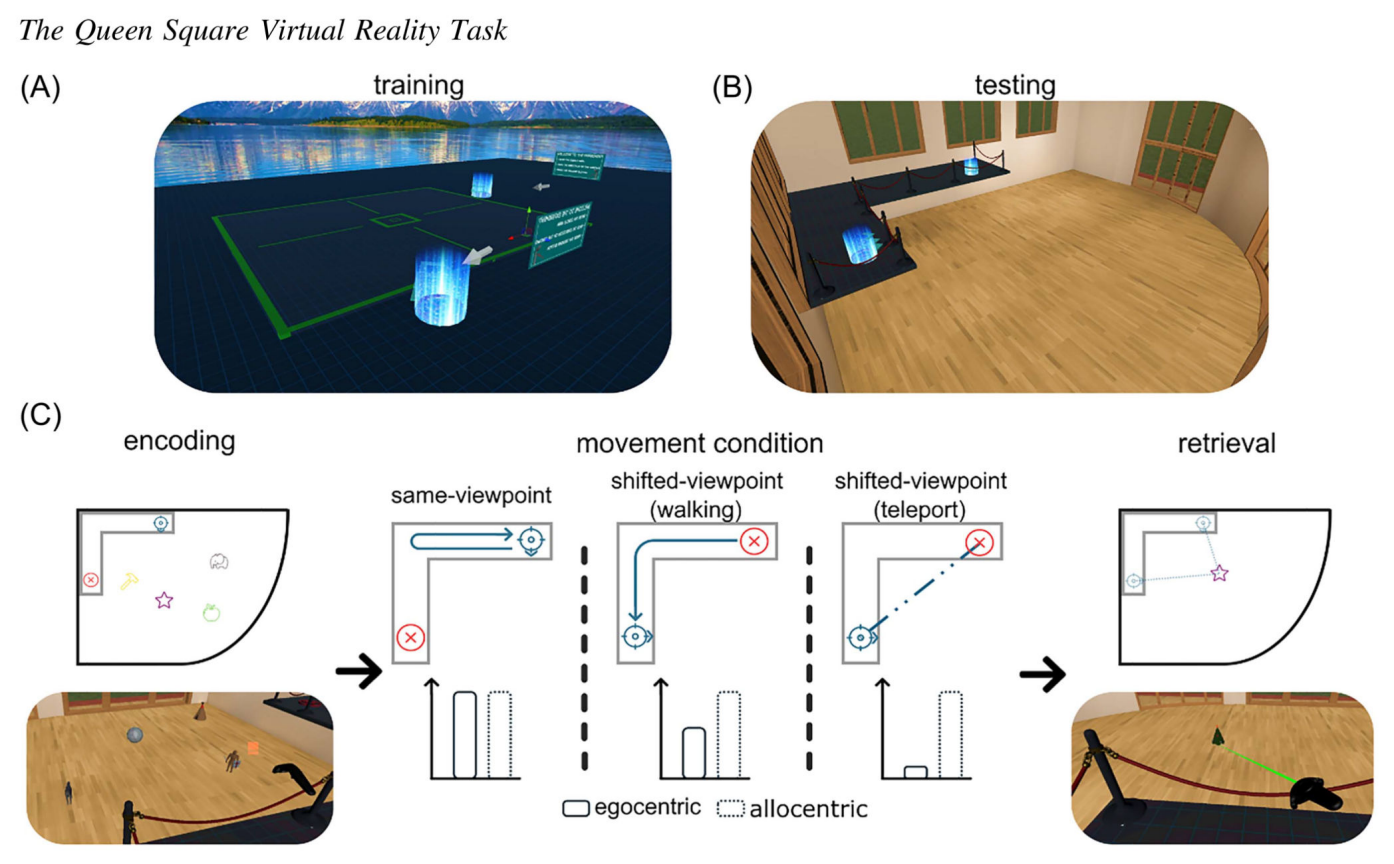
The Queen Square Virtual Reality Task *Note*. (A) Training phase environment. (B) Testing phase environment. The testing environment features a rectangular room with a rounded corner. Participants were positioned on an L-shaped platform, elevated 1.8 m above the floor, with a red rope barrier to mitigate vertigo. Two viewpoints on the platform, highlighted by blue lighting, were aligned with directional arrows to indicate the participants’ forward-facing orientation. (C) Schematic of the testing procedure. Trials began with participants at one of two viewpoints, counterbalanced across participants. During the encoding phase, either one or four objects were presented for 7 or 30 s in total. After encoding, the objects disappeared, and participants received instructions for one of three conditions: (a) Same-viewpoint—walking away from and back to the same viewpoint, which allows for the use of both egocentric (self-referenced) and allocentric (world-referenced) spatial representations; (b) shifted-viewpoint (walking)—walking to the other viewpoint, which encourages reliance on allocentric representations, as participants experience a change in perspective but still benefit from continuous self-motion updating of egocentric representations; (c) shifted-viewpoint (teleport)—teleporting to the other viewpoint using the controller, involving an automatic translation and rotation. This condition prevents continuous updating of egocentric representations by self-motion information, compelling participants to rely more heavily on allocentric representations to recall object locations. In the retrieval phase, participants used a virtual pointer to recall the locations of the objects, one at a time. Icons for the objects in part (C) are free resources from https://Flaticon.com. See the online article for the color version of this figure.

Participants were trained in a separate virtual environment (Figure 1A) to understand the task mechanics. They practiced memorizing an object’s location for 7 s before it disappeared, then replaced it using the controller’s trigger button as a “laser pointer,” repeated three times with feedback (these objects were not used in testing). Next, they practiced teleporting to “active” viewpoints using the controller’s thumbpad, with a quick fade to black screen transition (<~0.1 s) to mitigate motion sickness. Finally, they walked to different viewpoints following illuminated floor arrows, mirroring movement conditions in the tests. Participants could repeat any training steps before proceeding to the test phase (no training data were recorded).

### Testing Phase

The testing environment featured a rectangular room (15 m × 12 m) with a rounded corner and an elevated L-shaped platform (1.8 m above the floor and opposite to the rounded corner, Figure 1B), where participants stood throughout the task. Note that participants were able to walk freely on the platform. Two viewpoints were marked on the platform to guide participant movements and orientation, allowing for walking distances of 6.0 m and 4.5 m along the L-shaped platform. The maximum possible shifted-viewpoint rotation was 125°. The setup ensured that participants were able to actively navigate the space along the platform, engaging in natural self-motion during walking conditions.

At the start, each participant was assigned one of two viewpoints as the “same-viewpoint” in a counterbalanced design across participants. Consequently, the direction of the shifted-viewpoint transitions (i.e., from the same to the shifted viewpoint) remained consistent for each participant across all trials, and this directionality was counterbalanced between participants. Participants always began each trial from this same-viewpoint location. Each trial consisted of three steps (Figure 1C): (a) encoding, (b) movement manipulation, and (c) retrieval, where participants were asked to replace the objects in their original locations. Trials included either one or four objects.

Participants completed a total of 30 trials for each combination of movement condition and object configuration, divided into three blocks with optional 2-min rest periods between blocks.

The primary outcome measure was displacement error—the absolute distance in meters between the chosen and the actual object locations. The secondary outcome measure was retrieval time—the duration taken to place an object after receiving it via the virtual pointer.

### Encoding

Participants were instructed via in-VR messages to memorize the locations of either one or four objects placed at pseudo-randomized coordinates on the floor. They had 10 s for single-object trials and 30 s for four-object trials, with a timer displayed in the VR interface. After the allotted time, the objects and timer disappeared. Objects were placed at least 0.5 m apart to avoid overlap and occlusion.

Objects were selected from a pool of 108 unique low-poly 3D models of everyday items (e.g., fruits, furniture, animals, plants, utensils), each scaled to fit within 0.8 m^3^. No objects were repeated across trials.

### Movement Condition

Each trial included a movement manipulation (Figure 1C; Supplemental Video, Castegnaro et al., 2025): (a) “same-viewpoint” where participants walked along the platform’s side and returned to the starting viewpoint to control for the total amount of movement between encoding and retrieval; (b) “shifted-viewpoint (walking)” where participants walked to the other viewpoint, enabling continuous egocentric updating of spatial relationships; (c) “shifted-viewpoint (teleport)” where participants teleported, using the controller thumbpad button, to the other viewpoint, removing self-motion and environmental cues, thus requiring allocentric encoding due to disrupted egocentric cues. Note that the teleportation included both a virtual translation and rotation of the participants’ view. The environment remained visible throughout all movement conditions and participants’ walking trajectories were guided by illuminated directional arrows placed on the elevated L-shaped platform (see Supplemental Video). During the walking movement conditions, participants were instructed to keep their gaze aligned with the arrows on the elevated platform in order to discourage any strategy of maintaining a constant visual reference to the object’s previous location while walking. To minimize differences in exposure durations across movement conditions, we deliberately included a delay of 15 s from when the object disappeared at the end of the encoding phase to when the first object was presented back to participants during the retrieval phase. This duration was determined based on a conservative estimate of average walking speed (0.8 m/s) for older adults to traverse the maximum required distance (~12 m back and forth along the platform). This design decision ensured that even in the teleportation condition, participants would experience a minimum exposure time comparable to the walking conditions.

### Retrieval

After the movement condition, participants were prompted to replace the previously seen objects. A virtual laser pointer was activated, with a randomly selected object from the encoding phase appearing at its tip (Figure 1C). Participants aimed the laser at the desired location and confirmed placement with the trigger button; only one attempt was allowed per object. Objects were presented one at a time, and replacement was entirely self-paced (no time limit was imposed on the retrieval response).

### Neuropsychological Tests

To ensure that any cognitive impairments observed were within normal limits for aging, older participants underwent the Addenbrooke’s Cognitive Examination III. Only those scoring above the established threshold of 88 out of 100 were included in the study. Additionally, to facilitate comparisons between different testing modalities, older participants were also assessed using a desktop version of an allocentric spatial memory test, commonly known as the Four Mountains Test (Hartley et al., 2007). This allowed us to directly compare performances on a traditional desktop test proven to be hippocampal-dependent with those on the immersive virtual reality test of allocentric spatial memory.

### Analysis

Preprocessing, visualization and analysis were performed using Matlab Version 2020b. ANOVA analysis was conducted using SPSS.

### Object Location Memory Performance

Prior to running parametric tests, all continuous variables were checked for normality assumptions. Normality was assessed using the Shapiro–Wilk test (Shapiro & Wilk, 1965; suitable for distributions with *n* < 100 data points), alongside analyses of skewness and kurtosis. For the mixed-design ANOVA, which explored the effects of movement conditions and object configurations between young and older controls, the Levene test confirmed equality of variances, followed by Mauchly’s test of sphericity to check the assumption of sphericity. Due to a temporary tracking system fault, five trials from five participants in the older group were excluded.

### Factors Influencing Memory for Object Locations Within Different Movement Conditions and Within Different Configurations

A mixed ANOVA design was used to assess the effects of movement condition and object configuration on average displacement error, incorporating a three-way factor analysis within groups (movement condition, object configuration, block number) and a between-group factor of age (young and older adults). Analysis result for the block effect of the mixed ANOVA can be found in the Supplemental Materials. Planned within-group contrasts of the movement condition were performed using Helmert coding, allowing for comparisons between the average displacement errors of the same-viewpoint condition and the combined shifted-viewpoint conditions (across walking and teleporting), and then specifically between the two shifted-viewpoint conditions (walking vs. teleporting). Planned contrast on the different movement conditions within groups have been conducted a simple contrast coding. With this analysis choice the question asked, according to our hypothesis, was (a) does movement to different viewpoints affect memory for object locations? and (b) does a continuous spatial update of the one’s movement positively affect the retrieval for object locations from shifted viewpoints and does this benefit affect differently older individuals compared to younger ones?

To assess potential ceiling effects in performance in each movement condition, reflecting flooring effects in displacement errors, we conducted an analysis of error distributions, including measures of skewness, clustering near the theoretical minimum, and statistical comparisons against the floor theoretical minimum (see Supplemental Analysis). To ensure that skewed distributions did not influence the statistical comparisons, we applied a log transformation to displacement errors (see Supplemental Analysis) before running the mixed ANOVA analysis.

### Association With Comparative Allocentric Memory Test

An explorative analysis looked at the relation between the Four Mountains Test (4MT) score, which is a test of allocentric processing (Hartley et al., 2007), and the Queen Square VR displacement errors. Specifically, separate linear model has been fitted where the average displacement error per participant in each movement condition is the dependent variable and the score in the four mountains test is the independent variable. Outliers effect was reduced using a robust linear regression where the least-squares fitting is weighted usinga Tukey’s bisquare on each data point. A Bonferroni correction was applied to account for multiple comparisons. Higher scores in 4MT are expected to be correlated to lower displacement errors in the allocentric conditions of the Queen Square VR task.

### Transparency and Openness

All anonymized data, Matlab scripts, SPSS outputs used to produce the analysis and figures in this study have been made publicly available on the Github repository entitled QueenSquareVRAnalysis (Castegnaro et al., 2025). Please note these materials were not included in the peer-review process. Additional material is a video showcasing the three types of trials present in the study which is also been uploaded to said repository. There are no additional materials to share. The study design, hypotheses, and analytic plan were not preregistered.

## Results

### Differential Impact of Walking Versus Teleporting in Shifted-Viewpoint Conditions Between Age Groups

**Figure 2:**
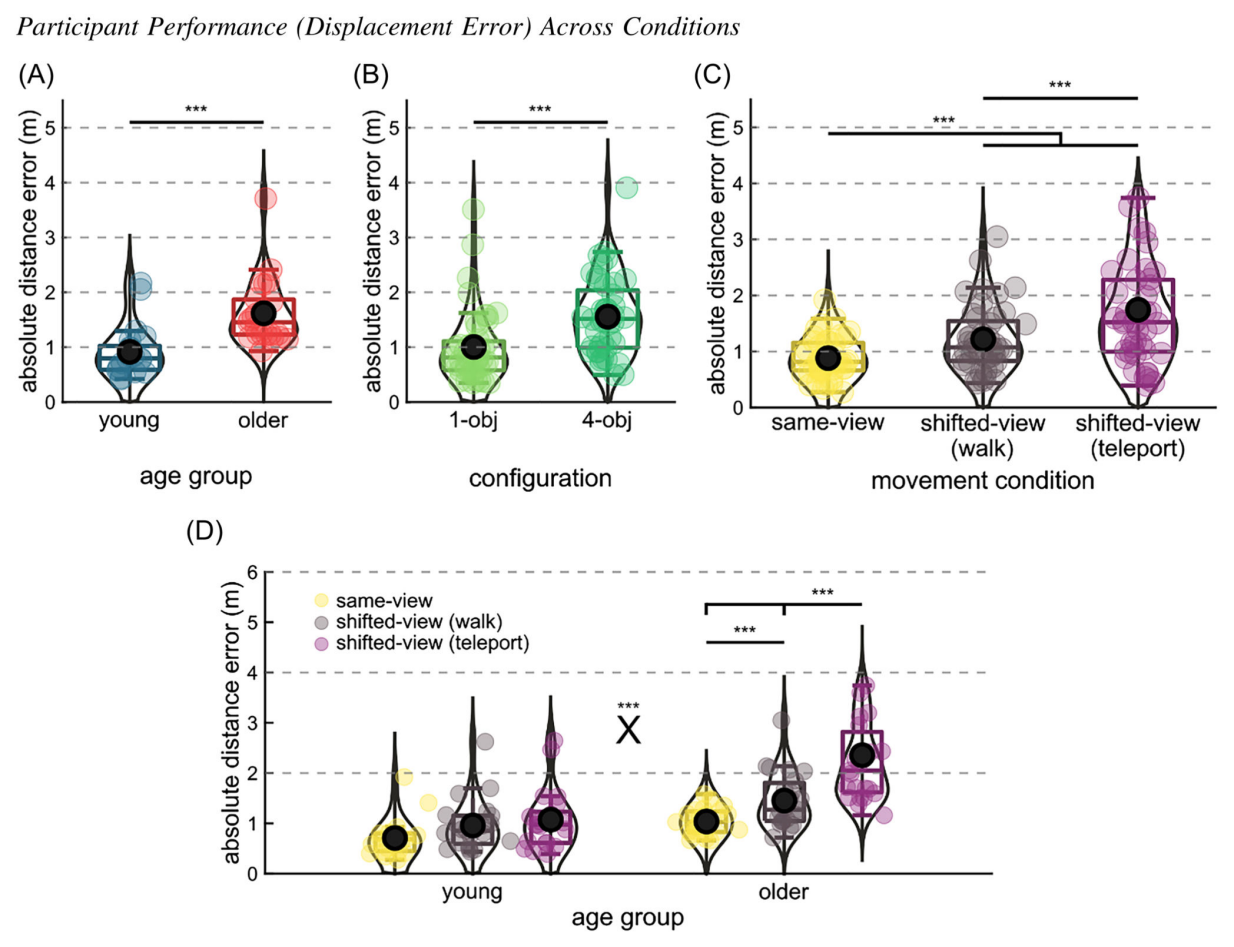
Participant Performance (Displacement Error) Across Conditions *Note*. All data are averaged over specified variables: (A) Performance by age group (young, older), averaged across object configurations and movement conditions. (B) Performance by object configuration (one or four objects), averaged across age groups and movement conditions. (C) Performance by movement condition, averaged across age groups and object configurations. (D) Performance by age group and movement condition, averaged across object configurations. Each violin plot was generated using kernel density estimation to show the probability density of the data at different values. Each circle represents the averaged displacement error for each participant. In each box, the large black dot marks the overall mean, and the dark grey bars mark the median and the 25th and 75th percentiles. Observations beyond the whisker length are outliers. A mixed analysis of variance revealed main effects of age group (A), object configuration (B) and movement condition (C) on displacement error. Planned contrast analysis revealed better performance across age groups in the same-view condition compared to the combined shifted-viewpoint conditions (C), and within shifted-viewpoint conditions, walking revealed better performance than teleporting (C) and no interaction between block and participant group. An interaction effect between movement condition and age group reveals that movement conditions did not affect the young group’s performance, while the older group performed best in the same-view condition, better in shifted-viewpoint when walking instead of teleporting (X symbol, D). See the online article for the color version of this figure. *** *p* < .001.

We observed significant main effects on average displacement error due to age group: *F*(1, 42) = 18.07, *p* < .001, = 0.30; Figure 2A, object configuration: *F*(1, 42) = 63.95, *p* < .001, ${\text{η}}_{\text{p}}^{2}=0.60$; Figure 2B, and movement condition: *F*(2, 41) = 34.25, *p* < .001, ${\text{η}}_{\text{p}}^{2}=0.45$. A nonsignificant trend toward a main effect of block number was observed (*p* = .53, see Supplemental Results for details). The older group performed worse (*M* = 1.61 m, *SD* = 0.61 m) than the young group (*M* = 0.91 m, *SD* = 0.46 m) across all conditions. Performance was poorer in the four-object configuration (*M* = 1.55 m, *SD* = 0.70 m) compared to the single object (*M* = 1.00 m, *SD* = 0.66 m).

Helmert planned contrasts indicated better performance in the same-viewpoint condition (*M* = 0.88 m, *SD* = 0.36 m) compared to combined shifted-viewpoint conditions, *M* = 1.47 m, *SD* = 0.93 m; *F*(1, 42) = 40.04, *p* < .001, ${\text{η}}_{\text{p}}^{2}=0.48$; Figure 2C, and within shifted-viewpoint conditions, walking (*M* = 1.22 m, *SD* = 0.58 m) out-performed teleporting, *M* = 1.74 m, *SD* = 1.12 m; *F*(1, 42) = 26.53, *p* < .001, ${\text{η}}_{\text{p}}^{2}=0.38$; Figure 2C.

There was a significant interaction effect between movement condition and age group, *F*(2, 41) = 12.91, *p* < .001, ${\text{η}}_{\text{p}}^{2}=0.23$, indicating differential impacts by movement conditions across age groups. Planned contrasts of the interaction effects revealed significant performance differences among older participants. Specifically, they performed significantly better in the same-viewpoint condition (*M* = 1.04 m, *SD* = 0.26 m) compared to both shifted-viewpoint conditions: walking, *M* = 1.45 m, *SD* = 0.54 m, *t*(22) = −4.17, *p* < .001, ${\text{η}}_{\text{p}}^{2}=0.44$, and teleporting, *M* = 2.36, *SD* = 1.15 m, *t*(22) = −6.15, *p* < .001, ${\text{η}}_{\text{p}}^{2}=0.63$. In addition, within the shifted-viewpoint conditions, older participants performed better when walking compared to teleporting, *t*(22) = −6.16, *p* < .001, ${\text{η}}_{\text{p}}^{2}=0.63$. In contrast, the younger participants showed no significant differences between movement conditions (all *p*s > .05), indicating that movement conditions did not affect their performance.

The interaction between object configuration and age group was not statistically significant, *F*(1, 42) = 4.01, *p* = .052, ${\text{η}}_{\text{p}}^{2}=0.09$, suggesting that the cognitive demands associated with increased object configuration difficulty did not differ significantly between the two age groups. The three-way interaction of movement condition, age group, and object configuration was not significant, nor was the full four-way interaction including block number.

Direct assessment of potential ceiling effects revealed that while younger adults exhibited some clustering toward lower values, performance was not constrained by a ceiling effect (see Supplemental Results).

To ensure that skewed distributions did not influence the statistical comparisons, we applied a log transformation to displacement errors before re-running the mixed ANOVA (see Supplemental Results). All main effects remained significant of age group, object configuration and movement condition remained highly significant (all *p*’s < .001), with increased effect sizes after transformation (age group: ${\text{η}}_{\text{p}}^{2}=0.38$; movement condition: ${\text{η}}_{\text{p}}^{2}=0.60$). Importantly, the interaction between age group and movement condition remained significant (*p* < .001). Planned contrast analysis (Supplemental Results) further revealed that younger participants performed better in the same-view condition compared to both shifted-viewpoint conditions (*p* < .01) but showed no difference between walking and teleporting in the shifted-viewpoint conditions. Older adults maintained the same performance pattern observed in the original analysis.

### Chance Performance and Retrieval Time Analysis

To assess if findings were driven by random guessing, we conducted a Supplemental Analysis to determine chance performance levels (see Supplemental Materials for details). Using a bootstrapping approach, we established that both young and older adults performed well above chance levels across all movement conditions, including the shifted-viewpoint (teleport) condition (Supplemental Figure S1).

In addition, an exploratory analysis examined the relationship between retrieval time and displacement error (see Supplemental Materials for details). While retrieval time was significantly longer in older adults compared to young participants (Supplemental Figure S5A), regression analyses revealed no significant relationship between retrieval time and displacement error in either age group (Supplemental Figure S5B).

### Association With Desktop Version of Allocentric Spatial Memory in Older Adults

**Figure 3:**
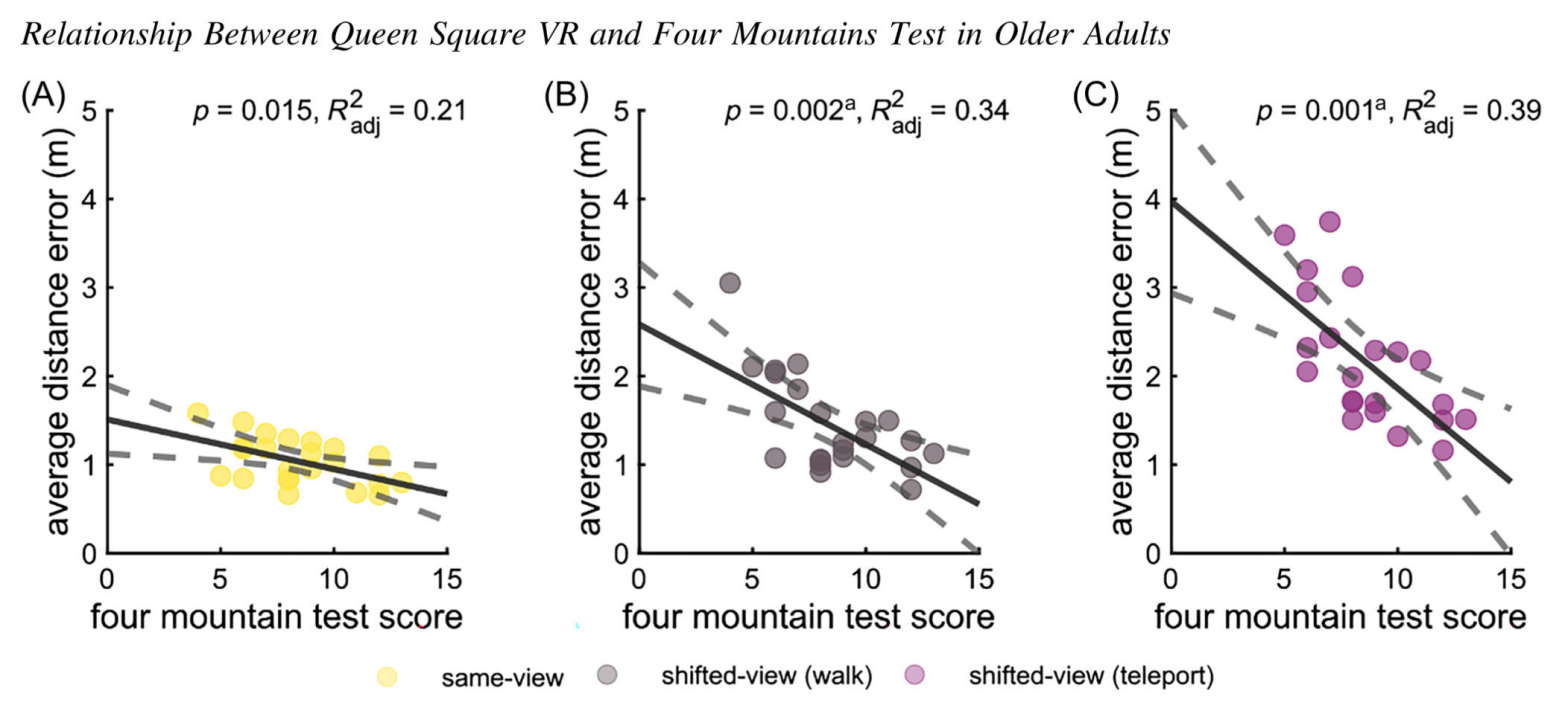
Relationship Between Queen Square VR and Four Mountains Test in Older Adults *Note*. Linear regression model examining the relationship between Four Mountains Test performance (*x*-axis)—measured as the number of correct answers—and Queen Square VR performance (*y*-axis) for older participants. Queen Square VR performance is represented by each participant’s average displacement error in the (A) same-viewpoint (B) shifted-viewpoint walking and (C) shifted-viewpoint teleport condition, averaged across all object configurations. The solid line represents the fitted regression, and the dashed lines indicate the 95% confidence intervals for the slope. Higher Four Mountains Test performance is associated with lower displacement error in the shifted-view walking (*p* = .002, *R*^2^_adjusted_ = 0.35) and shifted-viewpoint teleport conditions (*p* = .001, *R*^2^_adjusted_ = 0.39). VR = virtual reality. See the online article for the color version of this figure. ^a^ Statistically significant after Bonferroni correction for multiple comparisons.

Separate linear regression models were conducted for the older group to explore the relationship between average displacement error in each movement condition across object configurations of the Queen Square VR task and the 4MT scores. We did not collect 4MT data for younger adults as it was not part of the cognitive screening procedure to ensure that participants were cognitively normal. After adjusting for multiple comparisons, only the shifted-viewpoint teleport condition, *F*(1, 21) = 15.2, *p* = .001, *R*^2^_adjusted_ = 0.39; Figure 3C, and the shifted-viewpoint walking condition, *F*(1, 21) = 12.6, *p* = .002, *R*^2^_adjusted_ = 0.35; Figure 3B, showed significant negative associations with 4MT scores after Bonferroni correction, with the strongest effect observed in the teleport condition. For one unit increase of the 4MT performance the average displacement error decreased by 0.21 m in the shifted-viewpoint teleport condition while it decreased by 0.14 m in the shifted-viewpoint walking condition. The same-view condition approached significance but did not survive the multiple comparison correction (Figure 3A).

## Discussion

In this study, we investigated compensatory mechanisms in aging on spatial memory for object locations from different viewpoints using an immersive virtual reality task. Our main findings indicate that performance worsened in conditions requiring shifted viewpoints, particularly in the teleport condition, and this effect was primarily driven by the older participants. These findings suggest that the ability to update spatial representations through self-motion plays a critical role in mitigating age-related deficits in allocentric spatial memory.

This work builds on extensive evidence that advancing age is associated with deficits in spatial processing in tasks where a shift of the participant viewpoint is involved (Fernandez-Baizan et al., 2020; León et al., 2016; Tascón et al., 2019). In line with expectations, participants were most accurate at object replacement when using the same viewpoint. Consistent with our hypothesis, in the shifted-viewpoint conditions, walking resulted in significantly better object replacement compared to teleporting—where self-motion cues are removed (Figure 2C). While allocentric representations are available in all three conditions, an egocentric representation might better explain the pattern of results. In the same-view condition, both egocentric and allocentric representations are available, allowing participants to accurately recall object locations. In the shifted-viewpoint walking condition, participants can update their egocentric representations through vestibular input, optic flow, and proprioception to maintain spatial accuracy. However, in the shifted-viewpoint teleport condition, the instantaneous change in position and orientation prevents the updating of egocentric representations leaving participants to rely on allocentric representations to recall object locations. Although it is theoretically possible that participants could have engaged in imagined perspective-taking to maintain egocentric representations following teleportation, the cost associated with such mental transformations is known to increase sharply with angular disparity, peaking near 120°, which is closely matched by the ~125° shift in our teleport condition (Puls & May, 2020). In immersive VR, this cost is likely compounded by continuous sensory-motor input tied to participants’ real-world movements, which after teleportation may disrupt imagined realignment. Therefore, in the absence of continuous self-motion, participants were likely unable to rely on egocentric strategies alone.

Notably, while this pattern of decreased performance in shifted viewpoints was present across age groups (Supplemental Figure S3C)—reflecting the known baseline cost of perspective shifts even in young adults (Kelly & McNamara, 2010; Zancada-Menendez et al., 2016)—it was primarily driven by older participants (Figure 2D). The improved performance of older adults in the walking condition suggests that they effectively use self-motion information to update their egocentric representations, thereby compensating for weaker allocentric representations. In contrast, the absence of self-motion cues in the teleportation condition prevents this egocentric updating, leading older adults to rely solely on their weaker allocentric representations, resulting in increased errors.

Accounting for potential ceiling effects, particularly affecting performance in the young cohort, revealed that key effects not only remained significant but also showed stronger effect sizes (see Supplemental Results for details). Specifically, while both age groups showed performance decrements in shifted-viewpoint conditions, only older adults demonstrated a significant additional impairment when teleportation was used instead of walking (Supplemental Figure S3C). This pattern supports the interpretation that in shifted-viewpoint conditions, continuous self-motion information during walking provides significant benefits for spatial memory in older adults, enabling them to maintain better allocentric representations compared to teleportation. In contrast, younger adults can effectively compensate for the absence of such information during teleportation, exhibiting similar performance regardless of movement type when viewpoints change. Importantly, we ruled out that systematic directional biases induced by viewpoint shifts—previously identified in desktop VR and particularly pronounced in older participants (Segen et al., 2021)—could provide an alternative explanation for the age- and movement-related differences (see Supplemental Analysis). Specifically, our analysis (Supplemental Table S1) revealed no significant effects or interactions on projected directional errors, indicating that systematic directional biases were not present in this task and did not contribute to the observed pattern of increased errors among older adults in teleport compared to walking conditions (Supplemental Figure S4).

Previous work indicates that age-related allocentric decline is not uniform across paradigms (Hill et al., 2024; McAvan et al., 2021; Segen, Avraamides, et al., 2022). McAvan et al. (2021) showed that older adults can return accurately to a single hidden location in an ambulatory virtual Morris Water Maze; their task emphasizes path integration—updating the coordinates of a location that was previously visited—whereas our paradigm emphasizes spatial updating of one or more object locations relative to the broader environment without visiting those locations, a process that imposes a heavier representational load than tracking a single goal (Jahn et al., 2012; Wraga et al., 2000). In addition, McAvan et al. (2021) provided repeated training trials from multiple starting points before testing, whereas in our study each configuration had to be encoded on a single exposure and for a given time; the allocentric weakness we found might therefore be overcome when older adults can rehearse the layout (Castegnaro et al., 2022; Jansen et al., 2009; Sauzéon et al., 2016). Similarly, a desktop-VR viewpoint-shift study reported preserved accuracy in aging, yet the perspective rotation was 30°, smaller than the ~90–145° shifts that reveal allocentric deficits in the present experiment and in other work (King et al., 2002; Muffato et al., 2019; Puls & May, 2020; Reinoso Medina et al., 2025; Zancada-Menendez et al., 2016). In addition, their task required a binary same/different judgment, whereas our task requires a continuous, metric reconstruction of the entire configuration. Because coarse categorical decisions can often be solved through recognition-based familiarity, such tasks might place weaker demands on allocentric recollection (Bastin & Van der Linden, 2005; Muffato et al., 2022). Together, we argue that allocentric decline is most evident when (a) multiple object/landmark relations must be encoded in a single shot and (b) the perspective shift exceeds a certain threshold at which mental-rotation or recognition strategies become taxing. Future studies should take these boundary conditions into account when designing and interpreting spatial memory tasks across the adult lifespan.

To our best knowledge, this study is the first to explicitly link the weakness in allocentric strategies in older adults to continuous self-motion updates of spatial relationships using virtual reality, highlighting spatial updating as a mechanism that can help older adults to compensate for allocentric decline. We also examined whether older adults’ longer retrieval times (Supplemental Figure S5A) reflected greater uncertainty or a different response strategy rather than broader cognitive decline. However, our Supplemental Analysis (Supplemental Figure S5B) found no significant relationship between retrieval time and displacement error in either age group. Processing-speed theory propose that a central factor in adult cognitive aging is a reduction in the speed with which basic processing operations can be carried out, manifesting as longer completion times across a wide variety of tasks (Salthouse, 1996; Salthouse & Ferrer-Caja, 2003). Thus, the prolonged retrieval times found in older adults likely reflect this domain-general slowing rather than higher uncertainty or different response strategies.

The difficulties found by the older adults in the walking condition may be reflected within the normal range of aging in the decline of functionalities of the entorhinal cortex (Stangl et al., 2018) within the medial temporal lobe which support intrinsic self-motion related computations (Hafting et al., 2005; Sargolini et al., 2006). In addition, in the teleportation condition, where self-motion cues are absent, participants must rely more on allocentric processing to recall object locations. This allocentric processing is supported by brain structures such as the hippocampus, parahippocampal cortex (Burgess et al., 2002; Maguire, Burgess, et al., 1998; Maguire, Frith, et al., 1998), and the retrosplenial cortex which plays a key role in shifted-viewpoint memory (Bicanski & Burgess, 2016; Lambrey et al., 2012; Mitchell et al., 2018). Age-related decline in these structures, evidenced by a reduced activation when performing allocentric processing in navigation (Antonova et al., 2009; Moffat et al., 2006) could explain the greater accuracy difference observed in older participants between the walking and teleportation conditions.

A potential confounding factor in the observed deficit among older participants in the shifted-viewpoint (walking) condition may be age-related deterioration in the vestibular system. This deterioration can divert attention from navigation tasks to balance and postural control (Arshad & Seemungal, 2016), potentially reducing the attentional resources available for spatial updating. Teleportation, especially when involving both translational and rotational body-based cues, can induce disorientation (Cherep et al., 2020) due to abrupt changes in both position and orientation. However, our analysis confirmed performance above random guessing for both age groups, including teleportation (Supplemental Figure S1). This confirms that older adults’ increased errors in teleportation were not due to random responses but instead reflect difficulties in recalling object locations when self-motion cues were absent. The presence of environmental boundaries in our VR environment likely mitigated disorientation, as boundaries have been shown to help maintain orientation during teleportation (Kelly et al., 2022). We did not find any improvement of performance over time (Supplemental Figure S2), indicating that participants could adjust to the VR environment after the training phase. In this study, we did not record tracking data from the head-mounted display, which could have provided proxies for balance and posture. Future studies should consider incorporating such measures to monitor vestibular function during VR tasks, thereby controlling for potential vestibular deficits.

Our findings regarding the differential effects of teleporting and walking-induced viewpoint shifts in older adults may reflect distinctions between modality-dependent and modality-independent spatial representations (Huffman & Ekstrom, 2019; Steel et al., 2021). Modality-dependent spatial representations refer to spatial knowledge inherently tied to the sensory-motor modality through which it was originally encoded, requiring the reactivation of specific sensory-motor systems during spatial recall. Conversely, modality-independent, or amodal, spatial representations describe abstract cognitive maps that are not significantly influenced by the original encoding modality, allowing spatial knowledge to be expressed flexibly across different sensory modalities. In our study, older adults exhibited greater errors when teleporting disrupted the continuity of sensory-motor experiences compared to viewpoint shifts involving walking, consistent with an embodied cognition perspective emphasizing the integration of multimodal sensory information during spatial navigation. Previous studies indicate that multimodal sensory inputs significantly contribute to the formation of coherent spatial representations, particularly aiding in allocentric spatial memory performance in aged populations (Hill et al., 2024; McAvan et al., 2021) and in recruiting extrahippocampal strategies as shown in hippocampal lesion patients (Iggena et al., 2023). The instantaneous shift in viewpoint due to teleporting likely interrupts the integration of these multimodal cues, necessitating reliance on more abstract, amodal representations, which are less effectively maintained by older adults. This suggests not only that multimodal inputs enhance spatial memory performance, particularly under limited cognitive resources associated with aging (Huffman & Ekstrom, 2019), but also that these sensory inputs naturally form a unified representation in the brain, which is vulnerable to instantaneous discontinuities such as those introduced by teleporting (Steel et al., 2021). In contrast, younger participants, who typically show more efficient integration of sensory-motor information, exhibited no performance differences between teleporting and walking conditions, reflecting a resilience in both ego-centric spatial updating and the modality-independent representation of spatial layouts. These findings underline the importance of multimodal integration in spatial memory and suggest that age-related declines may impair the ability to flexibly shift between modality-dependent and modality-independent spatial representations.

Older adults’ performance on the Queen Square VR task was strongly correlated with performance on the 4MT, a hippocampal-dependent allocentric spatial memory assessment requiring landscapes to be recognized from a shifted viewpoint on the basis of topographical layout rather than visual appearance (see Figure 3; Hartley et al., 2007). Notably, both shifted-viewpoint conditions showed significant associations with 4MT scores, but the strongest effect was observed in the teleport condition. This finding highlights the link between allocentric spatial processing and performance in the Queen Square VR task, suggesting that the teleport condition most effectively isolates allocentric demands when self-motion cues are removed. These results support the association between age-related deficits, hippocampal function and suggest how the task proposed in this study supports amodal representations related to allocentric cognitive processing.

The higher cognitive load associated with remembering multiple object locations was found in the difference in performance (Figure 1B) between different object configuration, however, both young and older participants struggled similarly when memorizing four-object locations compared to one. This suggests that within our cohort, age-related decline did not significantly impact the ability to work with multiple objects within a scene, and the observed differences in movement conditions were not due to more general age-related declines in working memory capacity (Castillo Escamilla et al., 2023).

Impairments in allocentric processing (Serino et al., 2014) and path integration (Segen, Ying, et al., 2022) have been observed early in the progression to Alzheimer’s dementia. Thus, future research could employ shifted-viewpoint tasks that assess both allocentric and self-motion related processing to investigate which is specifically impaired in patients with mild cognitive impairment with an incipient cause of Alzheimer’s dementia. Since the anterolateral entorhinal cortex—a region implicated early in neurodegeneration (Braak & Del Tredici, 2015)—is critical for processing object locations within contexts (Olsen et al., 2017; Yeung et al., 2019), deficits in self-motion-related updating might be instrumental in stratifying patients according to Alzheimer’s dementia progression.

The present study has limitations. The sample size, though powered for detecting medium-sized effects, could have masked ceiling effects among younger participants or nonnormal distributions, potentially influencing the robustness and generalizability of our statistical analyses. Another limitation is the absence of precise timing data for each movement phase that could reveal extra time spent by participants re-orienting themselves—particularly after teleportation. We enforced a fixed minimum time between encoding and retrieval and we did not impose any upper limit on object replacement, ensuring each participant had an equal opportunity to reorient before placing the objects. Since retrieval time was not associated with displacement error in either age group (Supplemental Figure S5B), it is unlikely that faster or longer exploratory scanning could account for the observed age differences. Nonetheless, without continuous head-mounted display tracking we cannot exclude the possibility that younger adults engaged in more extensive visual scanning after teleportation—potentially allowing them to reacquire spatial landmarks more effectively and re-establish their environmental orientation. Future work should systematically integrate head-mounted display tracking to disentangle scanning behaviors from memory performance. In line with the previous point, the absence of head-mounted display tracking data could have directly confirmed participants’ head orientation throughout the task, particularly during walking movements. Such data would be necessary to assess whether participants attempted to hold a constant visual reference to the previous object location—thereby reducing the need for spatial updating via self-motion cues. Although our design features, including directional arrows and the spatially scattered object configurations, likely reduced this behavior, future research would benefit from incorporating tracking to quantify it.

In conclusion, the Queen Square VR task effectively detects age-related declines in spatial memory, with older adults showing significant impairments in the shifted-viewpoint tasks, and specifically when updating of egocentric representations via self-motion cannot be used to compensate for weak allocentric representation. These findings highlight the role of spatial updating in mitigating spatial memory deficits in aging.

## Public Significance Statement

This study identifies spatial updating as a compensatory mechanism in aging for mitigating allocentric spatial deficits. This mechanism is a potential target for early detection of Alzheimer’s dementia, as it involves key brain regions affected early in the disease.

## Footnotes

*Supplemental materials:* https://doi.org/10.1037/pag0000926.supp
